# Low levels of anti-MPER antibodies are detectable in viremic HIV infected

**DOI:** 10.1186/1742-4690-9-S2-P81

**Published:** 2012-09-13

**Authors:** J Carrillo, M Massanella, S MArfil, E Garcia, B Clotet, J Blanco

**Affiliations:** 1 IrsiCaixa/HIVACAT, Badalona, Spain

## Background

Antibodies against the CD4 binding site (CD4bs) in gp120 and the membrane proximal extracellular region (MPER) of gp41 are associated with broadly neutralization capacity. While the former have been identified in a large number of HIV infected individuals, the latter show a much lower prevalence.

## Methods

31 HIV-infected individuals with detectable viremia were selected for the study. Two samples separated by at least one year were analyzed for each individual. The presence of anti-CD4bs was screened using a competitive flow cytometric assay with a CD4 IgG fusion protein. The presence of anti-MPER antibodies was screened using a sensitive flow cytometric assay that measures antibody binding to different cell lines stably expressing two different truncated forms of gp41. These molecules properly expose the MPER epitope, as assessed by staining with control antibodies 4E10 and 2F5.

## Results

Detectable levels of both anti-CD4bs antibodies and anti-MPER antibodies were observed in plasma samples from all groups. Of note, most samples showed recognition of MPER with a strong correlation between the recognition of the two different forms of truncated gp41 used (r=0.65, p<0.0001), suggesting that the assay was robust enough for the detection of these antibodies. However, no correlations were found between the level of anti-MPER antibodies, the neutralizing capacity of plasma samples, the viral load and the CD4 T-cell counts.

## Conclusion

Anti-MPER antibodies can be detected in viremic chronic HIV infected individuals. The level of these antibodies does not appear to correlate with control of viremia or clinical progression. These data may suggest that anti-MPER antibodies are elicited in the course of HIV infection, but they do not reach the necessary threshold to be easily detectable or to impact infection.

